# Application of the Analgesia Nociception Index and Visual Analog Scale to Assess Pain During Hysteroscopy Under Local Anesthesia

**DOI:** 10.3390/jcm14238386

**Published:** 2025-11-26

**Authors:** Adrian Nowak, Karolina Chmaj-Wierzchowska, Adam Malinger, Maciej Wilczak

**Affiliations:** Department of Maternal and Child Health and Minimally Invasive Surgery, Poznan University of Medical Sciences, 60-701 Poznan, Poland; adam.malinger@ump.edu.pl (A.M.); mwil@ump.edu.pl (M.W.)

**Keywords:** minihysteroscopy 1, pain 2, visual analog scale (VAS) 3, analgesia nociception index (ANI)

## Abstract

**Background/Objectives**: Pain assessment during hysteroscopy facilitates rapid operator intervention (e.g., repeated anesthesia, administration of additional analgesics, or termination of the procedure), thereby improving patient comfort. Traditionally, pain intensity has been evaluated based on the patient’s subjective reporting; however, the introduction of an objective indicator in combination with subjective measures can considerably improve pain assessment. In this context, the present study aimed to evaluate pain intensity in patients undergoing hysteroscopy under local anesthesia (GUBBINI Mini Hystero-Resectoscope; Tontarra Medizintechnik, Tuttlingen, Germany), with simultaneous assessment using the Visual Analog Scale (VAS) and the Analgesia Nociception Index (ANI). Determining the relationship between ANI and VAS could contribute to improving patient comfort during hysteroscopic procedures performed under local anesthesia. **Methods**: The study included a total of 221 patients between the ages of 22 and 82 years, divided into two groups: 125 patients underwent diagnostic hysteroscopy (HD) and 96 patients underwent operative hysteroscopy (HO). After the procedure, patients were asked to assess pain intensity by using the VAS. The ANI was also monitored during the procedure. Measurements were conducted immediately before the procedure (T0), at the 1st minute (T1), mid-procedure (T1/2), and at the end of the procedure (T2). **Results**: The mean pain score in the study group was 3 points (3.04 ± 2.29), with a mean post-procedure score of 2.79 ± 2.19 and 3.36 ± 2.38 in the DH and OH groups, respectively. At Tmean, the ANI values ranged from 42 to 97 points, with a mean value of 70 points (70.37 ± 10.99). All correlations between the ANI values and VAS pain scores were statistically nonsignificant (*p* > 0.05), with correlation coefficients close to zero. This finding indicates that subjective pain assessment (VAS) does not correspond to the ANI values, which reflects the physiological autonomic response. **Conclusions**: Further research on pain assessment during hysteroscopy is warranted. Future studies should include larger patient populations, conduct continuous ANI monitoring, and correlate ANI with real-time subjective pain assessments (e.g., VAS during the procedure). The determination of psychological factors, such as pre-procedural anxiety, and the use of additional autonomic nervous system measures (e.g., skin conductance and pupillometry) could facilitate the development of a comprehensive model for objective pain assessment in patients undergoing gynecological procedures through hysteroscopy.

## 1. Introduction

Hysteroscopy is a minimally invasive endoscopic surgical method. Following its widespread use in the 1980s, it has been recognized as a diagnostic and therapeutic standard [1]. Hysteroscopy has broad clinical applications, and it can be used to evaluate abnormal uterine bleeding of unknown etiology, guide infertility management, and facilitate the treatment of intrauterine pathologies such as polyps, fibroids, adhesions, and even uterine septa. Two main types of hysteroscopic procedures are distinguished: diagnostic hysteroscopy (HD) and operative hysteroscopy (HO). Diagnostic hysteroscopy allows direct visualization of the cervical canal, uterine cavity, and tubal ostia, and, when necessary, enables targeted endometrial biopsy. Operative hysteroscopy, on the other hand, is used to remove lesions from the uterine cavity and/or cervical canal using mechanical, electrosurgical, or laser techniques [2,3,4,5,6]. Hysteroscopic procedures have a low complication rate (0–1.5%), while their diagnostic and therapeutic effectiveness is approximately 95% [7,8,9,10]. These data confirm the significance of hysteroscopy in contemporary medicine and establish it as a crucial tool in minimally invasive operative gynecology.

In spite of technological advances, pain experienced by patients during the procedure continues to be considered the main challenge. Previously, instruments with a diameter of 8–10 mm were used, requiring the use of a speculum, tenaculum, and gradual cervical dilation before inserting the endoscope, which typically necessitated general anesthesia [11,12]. The introduction of thinner resectoscopes, such as the GUBBINI system with a diameter of 5–6 mm, made it possible to perform procedures without cervical dilation and under local anesthesia [13]. The miniaturization of surgical instruments has improved procedural safety and tolerability, reduced hospitalization time, and lowered overall costs [14,15].

Despite sophisticated instruments and enhanced tolerance of hysteroscopy by patients, the procedure is still considered painful. Elevated pain intensity during the procedure contributes to a high incidence of vasovagal reactions, including fainting and hypotension, which may eventually require termination of the procedure. The prevalence of such events is 1.3–5.2% of the initiated procedures [16], making pain the most frequent reason for discontinuation. Although this is a common issue, there is yet no clear, standardized, and universally accepted protocol for using analgesics or anesthesia during hysteroscopy. Various approaches are available to completely eliminate the requirement for analgesia, such as delivery of local anesthesia to the cervix and administration of nonsteroidal anti-inflammatory drugs. However, because of lack of consensus, it is crucial to develop an appropriate strategy for both pharmacological management and procedural techniques

Pain assessment during hysteroscopy facilitates rapid operator intervention (e.g., repeated anesthesia, administration of additional analgesics, or termination of the procedure), thereby improving patient comfort. Notably, patients who do not experience pain during hysteroscopy cooperate better with the physician, which further translates into procedural success. Reliable pain assessment has cognitive and scientific significance. Traditionally, pain intensity has been evaluated based on the patient’s subjective reporting; however, the introduction of an objective indicator in combination with subjective measures can considerably improve pain assessment [17].

The Analgesia Nociception Index (ANI), based on the analysis of heart rate variability (HRV), has so far been primarily used to monitor the level of analgesia in patients under general anesthesia. Its applicability in conscious patients remains not fully understood. In a study by Issa et al., conducted in healthy volunteers, no significant correlation was found between ANI values and pain intensity assessed using the Numerical Rating Scale (NRS), indicating the need for further research on the usefulness of ANI in conscious settings [18]. Therefore, we aimed to evaluate the value of this index in patients undergoing hysteroscopy under local anesthesia, where an objective physiological response can be compared directly with the subjective perception of pain.

In this context, the present study aimed to evaluate pain intensity in patients undergoing hysteroscopy under local anesthesia, with simultaneous assessment using the Visual Analog Scale (VAS) and the Analgesia Nociception Index (ANI). Determining the relationship between ANI and VAS could contribute to improving patient comfort during hysteroscopic procedures performed under local anesthesia. It was assumed that there is a significant correlation between the Analgesia Nociception Index (ANI) values and the subjective pain scores on the Visual Analog Scale (VAS), and that ANI may serve as an objective indicator of the nociceptive response in conscious patients undergoing hysteroscopy.

## 2. Materials and Methods

This study was conducted at the Center for Hysteroscopy Under Local Anesthesia at the Heliodor Święcicki Gynecology and Obstetrics University Hospital, affiliated with the Poznań University of Medical Sciences, and enrolled 221 patients scheduled to undergo hysteroscopic treatment under local (paracervical, lidocaine-based) anesthesia. The Hysteroscopy Center is the first certified “CENTER OF EXCELLENCE” of the International Society for Gynecologic Endoscopy in Poland.

Eligibility criteria for the hysteroscopy procedure included abnormal uterine bleeding, intrauterine lesions such as endometrial polyps or submucosal fibroids, endometrial hyperplasia, or endometrial thickening. Patients with no history of hypersensitivity to lidocaine or ketoprofen, those in the follicular phase of the menstrual cycle, and postmenopausal women were included. Patients with heavy uterine bleeding and vaginal and/or cervical infections were excluded.

Patients were classified into the diagnostic hysteroscopy (HD) and operative hysteroscopy (HO) groups based on a transvaginal ultrasound examination performed prior to the procedure. Patients who required evaluation and, if necessary, endometrial biopsy were qualified for diagnostic hysteroscopy, whereas those requiring removal of intrauterine lesions such as polyps or fibroids were assigned to the operative hysteroscopy group.

Medical history was collected from the enrolled patients, including age, body weight, height, previous surgical procedures, history of drug allergies, number and type of deliveries, number of miscarriages, previous cervical or endometrial procedures, and general health status. Following gynecological and transvaginal ultrasound examinations, patients provided informed consent to undergo the hysteroscopic procedure.

### 2.1. Procedure Description

Thirty minutes before minihysteroscopy, each patient was intravenously administered 100 mg of ketoprofen. Vital parameters (heart rate, blood pressure, oxygen saturation, and respiratory rate) were continuously monitored throughout the procedure.

Ten minutes before introducing the minihysteroscope into the cervical canal, 10 mL of 0.1% lidocaine solution was administered locally—5 mL each at two paracervical sites (at 4 o’clock and 8 o’clock positions), where large blood vessels are absent, which allowed safe induction of local anesthesia. The anesthetic agent was injected using a needle with a penetration depth of approximately 2 mm (Hystero-Block).

The hysteroscopy procedure was performed using the vaginoscopic technique, without a speculum or tenaculum. Patients were positioned in the classic lithotomy position. The uterine cavity was distended with 0.9% NaCl solution as the distension medium, under continuous flow and 120 mmHg pressure. The procedure was conducted using the GUBBINI Mini Hystero-Resectoscope system (TONTARRA Medizintechnik GmbH, Daimlerstraße 15, 78573 Wurmlingen, Germany).

After the procedure, patients were asked to assess pain intensity by using the VAS. The ANI was also monitored during the procedure. Measurements were conducted immediately before the procedure (T0), at the 1st minute (T1), mid-procedure (T1/2), and at the end of the procedure (T2).

#### 2.1.1. Pain Testing Methods: VAS

The VAS is the most widely used tool for pain assessment, and its scale ranges from 0 (no pain) to 10 (the worst imaginable pain). VAS can be used to assess acute pain, such as intraoperative and postoperative pain, and chronic pain. Major advantages of the VAS are ease of use for both patients and physicians as well as reproducibility. These features explain its frequent use in pain measurement studies, including hysteroscopy, to evaluate the most painful stages of the procedure or to compare anesthesia protocols. The main limitations of the VAS are the lack of continuous pain monitoring during the medical procedure and its subjective nature. Therefore, it is crucial to complement the VAS with an objective physiological indicator responsive to nociceptive stimuli [19].

#### 2.1.2. Pain Testing Methods: ANI

Following advances in pain research in modern medicine, objective pain assessment methods based on physiological parameters have been introduced, which are independent of patient awareness and subjective reporting. A frequently used objective indicator is the ANI, a noninvasive index derived from heart rate variability (HRV) analysis. It reflects the degree of parasympathetic activity at a particular time point [20].

ANI values range from 0 to 100. Lower values (e.g., below 50) may indicate increasing pain intensity and sympathetic nervous system dominance, which are characteristics of nociceptive stress. Higher values (close to 100) reflect predominance of the parasympathetic nervous system, associated with reduced pain perception and improved patient comfort. The ANI was primarily introduced into anesthesiology as a tool to continuously monitor the analgesia–nociception balance, allowing anesthesiologists to assess whether the administered anesthetic agent adequately suppresses nociceptive responses during surgery. A key advantage of the ANI is the real-time measurement of values, enabling the detection of short pain episodes and immediate intervention by the physician. However, ANI also has limitations. The values may be influenced by significant stress or anxiety, which can independently reduce parasympathetic tone regardless of pain perception, thereby affecting ANI readings. Additionally, medications that alter heart rate and rhythm, such as beta-blockers, atropine, or sedatives, may interfere with the results. The application of the ANI in conscious patients remains under investigation [21,22].

### 2.2. Statistical Analysis

Statistica software (Cloud Software Group, Inc., Fort Lauderdale, FL, USA, 2023; Data Science Workbench, version 14), Jamovi (The Jamovi Project, 2022; version 2.3), and Microsoft Excel (Microsoft Office, 2019; version 2205) were used for data analysis. The Shapiro–Wilk test was applied to evaluate variable distribution. The Mann–Whitney *U* test and the Kruskal–Wallis test with Dunn’s post hoc comparisons were used for intergroup analyses. ANI values at different time points were compared with the Wilcoxon signed-rank test. Spearman’s rank correlation test and the chi-square test of maximum likelihood (Chi^2^ NW) were utilized to examine relationships between variables. A *p*-value of <0.05 was considered statistically significant in all calculations.

## 3. Results

### 3.1. Group Characteristics

The age of the study patients ranged from 22 to 82 years. The mean age of the study population was 44 years (46.76 ± 12.06), with a mean age of 46.79 ± 11.08 and 46.72 ± 13.3 years in the HD and HO groups, respectively. Body mass index (BMI) of the patients ranged from 16.9 to 42 kg/m^2^. The mean BMI was 24.8 kg/m^2^ (25.79 ± 5.18), with a mean value of 25.88 ± 5.21 and 25.68 ± 5.17 kg/m^2^ in the HD and HO groups, respectively. The characteristics of the study group are presented in Table 1.

We did not find significant differences between the type of hysteroscopy performed and the presence of comorbidities or a history of prior curettage (abrasion). Moreover, no significant associations were observed between the type of hysteroscopy performed and age or BMI.

### 3.2. Type of Hysteroscopy and Obstetric History

Thirty-one patients (14.03%) reported a history of miscarriage, while vaginal delivery was achieved in 147 patients (66.52%). No significant associations were found between the type of hysteroscopy and the occurrence or number of miscarriages/vaginal deliveries. A history of cesarean section was reported by 46 patients (20.81%). Women who underwent operative hysteroscopy were significantly less likely to have had a cesarean section. Conversely, women who underwent diagnostic hysteroscopy had a significantly higher number of cesarean deliveries. Additionally, women who underwent operative hysteroscopy were significantly less likely to have children. Table 2 presents the obstetric history of the patients according to the number of pregnancies and miscarriages as well as vaginal and cesarean deliveries.

In the operative hysteroscopy group, patients who had never been pregnant or given birth were significantly more frequent, while cesarean deliveries were significantly scarce. Table 3 presents the type of hysteroscopy according to obstetric history.

### 3.3. Procedure Duration

The procedure duration among all patients ranged from 9 to 45 min (Table 4). The mean procedure duration in the study group was 22.59 ± 6.83 min, with an average duration of 22.35 ± 5.98 min in the diagnostic hysteroscopy (HD) group and 22.9 ± 7.83 min in the operative hysteroscopy (OH) group.

Significant differences in procedure duration were observed depending on the type of hysteroscopy (Table 5).

Additionally, a significant correlation was not observed between procedure duration and patient age or BMI. In a subgroup analysis of patients undergoing DH, a strong trend was observed indicating that procedure duration tended to decrease with an increasing number of vaginal deliveries (ρ = 0.75; *p* = 0.084). Although this result was not significant (*p* < 0.05), it suggests a relationship that warrants further investigation in a larger patient cohort.

### 3.4. VAS

The pain assessment score among the patients ranged from 0 to 10 points. Table 6 presents the post-procedure pain scores for the entire study group. The mean pain score in the study group was 3 points (3.04 ± 2.29), with a mean post-procedure score of 2.79 ± 2.19 and 3.36 ± 2.38 in the DH and OH groups, respectively.

Patients who underwent DH and OH showed no significant differences in pain intensity assessed using the VAS (*p* = 0.15), although a nonsignificant trend toward higher pain scores was noted in the OH group (Table 7).

VAS pain scores exhibited no significant correlation with patient age or BMI. In the subgroup of patients with OH, a strong trend was observed indicating that a higher number of previous cesarean sections was associated with lower pain scores (VAS). Although this result (τ = −0.91; *p* = 0.051) was not statistically significant (*p* < 0.05), it suggests a relationship worthy of further investigation.

### 3.5. ANI

The ANI values at T0 among the patients ranged from 18 to 100 points, with a mean value of 62 points (62.26 ± 15.2) in the study group. The ANI values at T1 ranged from 33 to 98 points, with a mean value of 69 points (67.14 ± 15.47). At T1/2, the ANI values ranged from 43 to 100 points, with a mean value of 74 points (73.33 ± 12.95). At T2, the ANI values ranged from 36 to 98 points, with a mean value of 70 points (69.06 ± 13.11). At Tmean, the ANI values ranged from 42 to 97 points, with a mean value of 70 points (70.37 ± 10.99). The ANI values at different time points are presented in Table 8.

Abnormal ANI values were observed in 47 patients (21.3%) at T0, 33 patients (14.9%) at T1, 7 patients (4.93%) at T1/2, and 14 patients (6.3%) at T2. ANI values at different time points depending on the type of hysteroscopy are presented in Table 9.

Furthermore (Table 10), the ANI values increased significantly during the procedure; the value was the lowest at T0 (mean 62.26 ± 15.2), and it became significantly higher at the subsequent time points of T1, T1/2, T2, and Tmean, as confirmed by the Wilcoxon signed-rank test (all *p* < 0.001, except T1 vs. T2: *p* = 0.13).

The T0 measurement had the lowest value, and all subsequent measurements showed significantly higher values compared to T0. The T1/2 measurement showed the highest value and was significantly higher than all other measurements. The values of T1 and T2 alone were similar to each other. Wilcoxon signed-rank test results between individual ANI measurements depending on the type of hysteroscopy are presented in Table 11.

**Figure 1:**
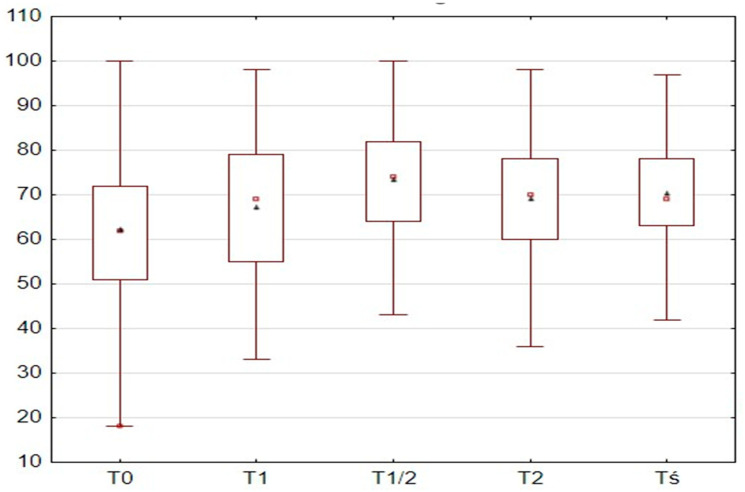
Comparison of ANI measurements (▫ mean and ∆ standard deviation).

As shown in Figure 1, significant differences were observed between the individual ANI measurements. The T0 measurement had the lowest value, and all subsequent measurements showed significantly higher values compared to T0. The T1/2 measurement showed the highest value and was significantly higher than all other measurements. The values of T1 and T2 alone were similar to each other.

Table 12 presents Spearman’s correlation coefficients (R) between the ANI values at different time points and age, procedure duration, BMI, and VAS scores. A relationship was observed only with patient age, i.e., the ANI values tended to decrease with an increasing age. No linear correlation was found between the ANI values and VAS scores.

Spearman correlation coefficients (R) between the ANI values at different time points and age, procedure duration, BMI, and VAS scores depending on the type of hysteroscopy are presented in Table 13.

In both DH and OH groups, higher patient age was associated with lower ANI values at Tś. This relationship was characterized by a significant, strong negative correlation in both groups, suggesting that age may influence the autonomic nervous system response regardless of the type of procedure performed.

Furthermore, the ANI values at T0 were significantly higher in patients undergoing OH than in those undergoing DH (*p* = 0.01; Table 14). No significant differences between groups were observed for subsequent measurements: T1, T1/2, T2, and Tmean (*p* > 0.05). Moreover, no significant differences were detected for the remaining measurements.

We also did not find a significant relationship between T1 values and the number of previous pregnancies in the DH group (ρ = 0.34; *p* = 0.45). In the OH group, no significant associations were observed between the number of previous cesarean sections and ANI measurements, including T0, T2, and Tmean, although trends suggested a possible relationship. The other ANI measurements also showed no correlations with the number of miscarriages, vaginal deliveries, or previous curettage procedures. The ANI values also did not correlate significantly with variables such as BMI, procedure duration, or pain assessment using the VAS.

T0 measurements were significantly higher in patients undergoing OH (*p* = 0.01). However, no significant differences between the groups were observed for the subsequent measurements (T1, T1/2, T2, and Tś). Additionally, the T0 ANI values were significantly lower in patients with comorbidities than in those without comorbidities (*p* = 0.01). No significant differences were observed for subsequent measurements.

### 3.6. Correlation Between the ANI and VAS

All correlations between the ANI values and VAS pain scores were statistically nonsignificant (*p* > 0.05), with correlation coefficients close to zero. This finding indicates that subjective pain assessment (VAS) does not correspond to the ANI values, which reflects the physiological autonomic response. Table 15 presents the VAS and ANI results. No relationship was observed between the type of hysteroscopy and the obtained scores.

As shown in Table 16, no relationship was observed between VAS scores and individual ANI measurements. Table 14 and Table 15 from the article present the relationships between VAS groups and ANI categories—there is no division by type of hysteroscopy and its introduction would be problematic due to the number of subjects. Without division, some categories contain 30 people per group, which is the minimum recommended number.

As shown in Table 17, no relationship was observed between individual ANI measurements and VAS scores.

## 4. Discussion

The present study aimed to analyze pain perception during the hysteroscopy procedure performed under local anesthesia by using both subjective assessment (VAS) and an objective measure of autonomic nervous system activity (ANI).

Pain experienced during the hysteroscopy procedure is subjective and influenced by factors such as the size and flexibility of the instruments used, procedure duration, operator experience, individual patient characteristics (e.g., cervical length, history of vaginal deliveries), type of gynecological abnormality, and the patient’s psychological profile. Therefore, understanding these variables and monitoring pain intensity is crucial for the operator to accordingly adjust the surgical technique and analgesic measures [2].

The results revealed that, despite mild pain reported by patients on the VAS, the ANI values significantly fluctuated throughout the procedure, suggesting that the objective physiological response to painful stimuli does not always correspond to the subjective experience reported by patients. Notably, the ANI values increased during the procedure and reached the highest mean level at mid-procedure (T1/2), which may reflect the effect of analgesics and autonomic adaptation to procedural stimuli.

The results of the VAS scores indicated a mean pain score of 3.04 ± 2.29, corresponding to moderate pain and supporting local anesthesia application as an effective analgesic method during hysteroscopic procedures. The absence of significant differences in pain levels between the DH and OH groups suggests comparable tolerability of both procedures, provided the analgesic preparation is adequate. Notably, in the OH group, a higher number of previous cesarean sections was associated with lower pain scores, potentially reflecting higher pain tolerance or anatomical changes in the cervix that reduce sensitivity to instrument manipulation.

Similarly, significant differences in the ANI values were observed between pre-procedure measurements (T0) and the subsequent procedural stages. The increase in the ANI values after the start of the procedure (T1) and further increase at T1/2 may indicate enhanced parasympathetic dominance and reflect the effectiveness of local anesthesia. Moreover, these values align with thresholds considered comfortable for intraoperative analgesia (ANI values above 60 indicate adequate pain control). Consistent with the meta-analysis of Baroni et al. as well as our study, no correlation was found between the VAS scores and the ANI values, suggesting that the ANI values in conscious patients may be influenced not only by painful stimuli but also by other factors such as emotional state [21].

With advancing age, a decrease in parasympathetic activity, reduced vagal tone, and diminished heart rate variability (HRV) are observed, along with a relative increase in sympathetic activity. These parameters are crucial for ANI values, as the index is based on the analysis of parasympathetic modulation. Therefore, in older patients, ANI values may be lower or less dynamic regardless of the actual intensity of pain. This mechanism may partially explain the relationship observed in our study between ANI and age, as well as the lack of correlation between ANI and subjectively reported pain intensity in conscious patients. An interesting finding was that higher patient age was associated with lower ANI values, which may reflect reduced parasympathetic activity, including decreased HRV, attenuated cardiac response to physiological stimuli, and increased sympathetic activity (higher vascular tone and blood pressure) [23].

Additionally, a higher number of previous pregnancies and cesarean sections was associated with higher ANI values at some measurement points (T0 and T2), which may indicate increased pain tolerance due to prior childbirth experience or anatomical and functional adaptations of the pelvic organs. These results are consistent with those of Zayed et al., who reported that multiparous women are more resistant to pain during hysteroscopy, and with the findings of DuBuc, who demonstrated that nulliparous women experience more pain during intrauterine device insertion [24,25].

A noteworthy finding is that a significant correlation was observed between procedure duration and ANI (T1) only in the DH group. Thus, longer procedures were associated with higher ANI values, suggesting gradually increasing analgesic effectiveness or physiological adaptation to stimuli, particularly in less invasive procedures.

The results of the present study are consistent with the observations of other authors who also did not confirm a significant correlation between ANI values and pain assessment in conscious patient populations. In the study by Issa et al. and in the meta-analysis by Baroni et al., ANI was shown to be a reliable indicator of analgesia in patients under general anesthesia; however, its usefulness in conscious conditions appears to be limited due to the influence of emotional factors and individual differences in autonomic nervous system activity [18,21]. Our findings support these observations, indicating that in patients undergoing hysteroscopy under local anesthesia, ANI does not reflect the subjective intensity of pain, and its clinical value requires further validation.

The absence of correlation between BMI and ANI or VAS suggests that body mass minimally affects pain perception and physiological response during hysteroscopy under local anesthesia.

### 4.1. Study Limitations

Several limitations of this study should be considered when interpreting the results. First, the ANI was measured only at discrete time points rather than continuously, which prevented detection of short-term pain or stress episodes. Second, the ANI is influenced by multiple variables, including emotional state, medications, general health, and even respiration, which complicates its interpretation, particularly in conscious patients. Third, subjective pain assessment scores (VAS) were collected only at the post-procedure stage, which introduces potential recall bias and does not reflect periodic pain. Finally, the limited sample size and single-center design restrict the generalizability of the findings.

### 4.2. Directions for Future Research

In the present study, ANI did not reflect the subjective perception of pain during hysteroscopy performed under local anesthesia. Consequently, the use of ANI as a clinical tool in conscious patients requires further validation.

Further research on pain assessment during hysteroscopy is warranted. Future studies should include larger patient populations, conduct continuous ANI monitoring, and correlate ANI with real-time subjective pain assessments (e.g., VAS during the procedure). The determination of psychological factors, such as pre-procedural anxiety, and the use of additional autonomic nervous system measures (e.g., skin conductance and pupillometry) could facilitate the development of a comprehensive model for objective pain assessment in patients undergoing gynecological procedures through hysteroscopy.

## Figures and Tables

**Table 1:** Characteristics of the study group.

		*n* = 221	%
Age (years)	<25	3	1.36%
	26–35	28	12.67%
	36–50	127	57.47%
	51–65	40	18.10%
	>65	23	10.40%
BMI (kg/m^2^)	Underweight—BMI < 20	22	9.95%
	Normal body weight—BMI: 20–24.9	92	41.63%
	Overweight—BMI: 25–29.9	65	29.41%
	Obesity—BMI: ≥30	42	19.01%
Comorbidities	Yes	141	63.8%
	No	80	36.2%
Abrasion	Yes	36	16.29%
	No	185	83.71%

**Table 2:** Type of hysteroscopy and the number of pregnancies and miscarriages as well as vaginal deliveries and cesarean sections.

		Type of Hysteroscopy		Total	χ^2^	*p*
		Diagnostic	Operative			
Pregnancies	0	12 (9.6%)	26 (27.08%)	38 (17.19%)	14.7	0.02
	1	31 (24.8%)	21 (21.88%)	52 (23.53%)		
	2	57 (45.6%)	36 (37.5%)	93 (42.08%)		
	3	20 (16%)	12 (12.5%)	32 (14.48%)		
	4	2 (1.6%)	0 (0%)	2 (0.9%)		
	5	1 (0.8%)	0 (0%)	1 (0.45%)		
	7	2 (1.6%)	1 (1.04%)	3 (1.36%)		
Miscarriages	0	103 (82.4%)	87 (90.63%)	190 (85.97%)	3.63	0.3
	1	19 (15.2%)	7 (7.29%)	26 (11.76%)		
	2	2 (1.6%)	1 (1.04%)	3 (1.36%)		
	3	1 (0.8%)	1 (1.04%)	2 (0.9%)		
Vaginal deliveries	0	36 (28.8%)	38 (39.58%)	74 (33.48%)	5.16	0.4
	1	32 (25.6%)	21 (21.88%)	53 (23.98%)		
	2	47 (37.6%)	30 (31.25%)	77 (34.84%)		
	3	8 (6.4%)	7 (7.29%)	15 (6.79%)		
	6	1 (0.8%)	0 (0%)	1 (0.45%)		
	7	1 (0.8%)	0 (0%)	1 (0.45%)		
Cesarean sections	0	93 (74.4%)	82 (85.42%)	175 (79.19%)	10.08	0.02
	1	22 (17.6%)	10 (10.42%)	32 (14.48%)		
	2	10 (8%)	2 (2.08%)	12 (5.43%)		
	3	0 (0%)	2 (2.08%)	2 (0.9%)		

**Table 3:** Type of hysteroscopy according to obstetric history.

		Type of Hysteroscopy		Total	χ^2^	*p*
		Diagnostic	Operative			
Pregnancies	Yes	113 (90.4%)	70 (72.92%)	183 (82.81%)	11.66	0.001
	No	12 (9.6%)	26 (27.08%)	38 (17.19%)		
Miscarriages	Yes	22 (17.6%)	9 (9.38%)	31 (14.03%)	3.16	0.08
	No	103 (82.4%)	87 (90.63%)	190 (85.97%)		
Vaginal deliveries	Yes	89 (71.2%)	58 (60.42%)	147 (66.52%)	2.82	0.09
	No	36 (28.8%)	38 (39.58%)	74 (33.48%)		
Cesarean sections	Yes	32 (25.6%)	14 (14.58%)	46 (20.81%)	4.11	0.04
	No	93 (74.4%)	82 (85.42%)	175 (79.19%)		
Parity	Nulliparous	16 (12.8%)	28 (29.17%)	44 (19.91%)	9.27	0.01
	Primiparous	35 (28%)	24 (25%)	59 (26.7%)		
	Multiparous	74 (59.2%)	44 (45.83%)	118 (53.39%)		
Preterm deliveries	Yes	4 (3.2%)	3 (3.13%)	7 (3.17%)	0	0.97
	No	121 (96.8%)	93 (96.88%)	214 (96.83%)		

**Table 4:** Hysteroscopy duration procedure time for the entire group.

		*n* = 221	%
Procedure duration (min)	<10	5	2.26%
	11–15	35	15.84%
	16–20	76	34.39%
	21–25	62	28.05%
	26–30	21	9.5%
	31–35	14	6.34%
	36–40	4	1.81%
	>41	4	1.81%

**Table 5:** Procedure duration depending on the type of hysteroscopy.

Procedure Duration (min)	Type of Hysteroscopy		Total	χ^2^	*p*
	Diagnostic	Operative			
<10	0 (0%)	1 (1.04%)	1 (0.45%)	21.75	0.003
10–15	15 (12%)	24 (25%)	39 (17.65%)		
16–20	52 (41.6%)	24 (25%)	76 (34.39%)		
21–25	42 (33.6%)	20 (20.83%)	62 (28.05%)		
26–30	7 (5.6%)	14 (14.58%)	21 (9.5%)		
31–35	5 (4%)	9 (9.38%)	14 (6.33%)		
36–40	2 (1.6%)	2 (2.08%)	4 (1.81%)		
>41	2 (1.6%)	2 (2.08%)	4 (1.81%)		

**Table 6:** VAS scores.

		*n* = 221	%
VAS	0	33	14.93%
	1	34	15.38%
	2	31	14.03%
	3	37	16.74%
	4	32	14.48%
	5	22	9.95%
	6	11	4.98%
	7	10	4.53%
	8	9	4.08%
	9	1	0.45%
	10	1	0.45%

**Table 7:** VAS scores depending on the type of hysteroscopy.

VAS	Type of Hysteroscopy		Total	χ^2^	*p*
	Diagnostic	Operative			
0	18 (14.4%)	15 (15.63%)	33 (14.93%)	14.61	0.15
1	24 (19.2%)	10 (10.42%)	34 (15.38%)		
2	23 (18.4%)	8 (8.33%)	31 (14.03%)		
3	19 (15.2%)	18 (18.75%)	37 (16.74%)		
4	14 (11.2%)	18 (18.75%)	32 (14.48%)		
5	11 (8.8%)	11 (11.46%)	22 (9.95%)		
6	7 (5.6%)	4 (4.17%)	11 (4.98%)		
7	4 (3.2%)	6 (6.25%)	10 (4.52%)		
8	4 (3.2%)	5 (5.21%)	9 (4.07%)		
9	1 (0.8%)	0 (0%)	1 (0.45%)		
10	0 (0%)	1 (1.04%)	1 (0.45%)		

**Table 8:** ANI values at different time points.

Time Point	ANI	Mean	M ± SD
T0	18–100	62	62.26 ± 15.2
T1	33–98	69	67.14 ± 15.47
T1/2	43–100	74	73.33 ± 12.95
T2	36–98	70	69.06 ± 13.11
Tmean	42–97	69	70.37 ± 10.99

T0—time before the procedure (time between administration of pericervical anesthesia and removal of the speculum and insertion of the hysteroscope into the vagina), T1—at the 1st minute of the hysteroscopy, T1/2—mid-procedure of the hysteroscopy, T2—time at the end of the procedure of the hysteroscopy, Tmean—average hysteroscopy procedure time.

**Table 9:** ANI values at different time points depending on the type of hysteroscopy.

	Type of Hysteroscopy						Total			U	***p***
	Diagnostic			Operative							
	M ± SD	Min-Max	Me [Q1–Q3]	M ± SD	Min-Max	Me [Q1–Q3]	M ±SD	Min-Max	Me [Q1–Q3]		
T0	59.73 ± 14.24	18–90	60 [50–70]	65.56 ± 15.83	35–100	65.5 [51–78.5]	62.26 ±15.2	18–100	62 [51–72]	4806.5	0.01
T1	66.37 ± 15.14	33–96	66 [55–78]	68.16 ± 15.91	34–98	70 [56–80.25]	67.14 ± 15.47	33–98	69 [55–79]	5553	0.34
T1/2	72.07 ± 13.14	45–99	72 [62–81]	74.97 ± 12.58	43–100	77 [66.75–83]	73.33 ± 12.95	43–100	74 [64–82]	5148	0.07
T2	69.38 ± 13.25	37–98	70 [60–78]	68.65 ± 12.98	36–96	6.5 [59.75–77.25]	69.06 ± 13.11	36–98	70 [60–78]	5854	0.76
T mean	70.5 ± 11.18	42–97	70 [63–79]	70.2 ± 10.78	42–96	69 [62.75–77.25]	70.37 ± 10.99	42–97	69 [63–78]	5908	0.85

T0—time before the procedure (time between administration of pericervical anesthesia and removal of the speculum and insertion of the hysteroscope into the vagina), T1—at the 1st minute of the hysteroscopy, T1/2—mid-procedure of the hysteroscopy, T2—time at the end of the procedure of the hysteroscopy, Tmean—average hysteroscopy procedure time.

**Table 10:** Wilcoxon signed-rank test results between individual ANI measurements.

	Paired Measurements			
	N	T	Z	*p*
T0 & T1	218	8718.00	3.45	0.001
T0 & T1/2	219	4568.50	7.96	<0.001
T0 & T2	216	7201.50	4.91	<0.001
T0 & Tmean	218	6066.50	6.29	<0.001
T1/2 & T1	216	7295.50	4.81	<0.001
T1/2 & T2	217	8042.50	4.09	<0.001
T1/2 & Tmean	215	8447.50	3.46	0.001
T1 & T2	214	10,134.50	1.51	0.13
T1 & Tmean	217	9410.50	2.61	0.01
T2 & Tmean	209	9045.00	2.20	0.03

T0—time before the procedure (time between administration of pericervical anesthesia and removal of the speculum and insertion of the hysteroscope into the vagina), T1—at the 1st minute of the hysteroscopy, T1/2—mid-procedure of the hysteroscopy, T2—time at the end of the procedure of the hysteroscopy, Tmean—average hysteroscopy procedure time.

**Table 11:** Wilcoxon signed-rank test results between individual ANI measurements depending on the type of hysteroscopy.

	Type of Hysteroscopy		Total
	Diagnostic	Operative	
T0 & T1	3.4 **	1.31	3.45 **
T0 & T1/2	6.49 ***	4.66 ***	7.96 ***
T0 & T2	5.24 ***	1.45	4.91 ***
T0 & Tś	6.29 ***	2.31 *	6.29 ***
T1 & T1/2	3.59 ***	3.21 **	4.81 ***
T1 & T2	1.96	0.01	1.51
T1 & Tś	2.65 *	0.91	2.61 *
T1/2 & T2	2.02 *	3.91 ***	4.09 ***
T1/2 & Tś	1.41	3.55 ***	3.46 **
T2 & Tś	1.66	1.49	2.2 *

T0—time before the procedure (time between administration of pericervical anesthesia and removal of the speculum and insertion of the hysteroscope into the vagina), T1—at the 1st minute of the hysteroscopy, T1/2—mid-procedure of the hysteroscopy, T2—time at the end of the procedure of the hysteroscopy, Tmean—average hysteroscopy procedure time. * *p* < 0.05; ** *p* < 0.01; *** *p* < 0.001.

**Table 12:** Spearman correlation coefficients (R) between the ANI values at different time points and age, procedure duration, BMI, and VAS scores.

	Age	Procedure Duration	BMI	VAS
T0	−0.16 *	−0.09	−0.08	0.08
T1	−0.06	−0.01	−0.04	−0.03
T1/2	−0.19 **	0.04	−0.09	−0.02
T2	−0.19 **	0	−0.07	−0.01
Tmean	−0.25 ***	0.01	−0.06	0

T0—time before the procedure (time between administration of pericervical anesthesia and removal of the speculum and insertion of the hysteroscope into the vagina), T1—at the 1st minute of the hysteroscopy, T1/2—mid-procedure of the hysteroscopy, T2—time at the end of the procedure of the hysteroscopy, Tmean—average hysteroscopy procedure time. * *p* < 0.05; ** *p* < 0.01; *** *p* < 0.001.

**Table 13:** Spearman correlation coefficients (R) between the ANI values at different time points and age, procedure duration, BMI, and VAS scores depending on the type of hysteroscopy.

Type of Hysteroscopy	ANI	Age	Procedure Duration	BMI	VAS
Diagnostic	T0	−0.07	−0.15	−0.06	0.09
	T1	−0.03	−0.14	0	−0.11
	T1/2	−0.25 **	−0.08	−0.06	−0.05
	T2	−0.24 **	−0.11	−0.09	−0.05
	Tmean	−0.28 **	−0.15	−0.05	0.06
Operative	T0	−0.24 *	−0.02	−0.12	0.04
	T1	−0.12	0.1	−0.09	0.03
	T1/2	−0.12	0.17	−0.14	−0.01
	T2	−0.14	0.12	−0.05	0.05
	Tmean	−0.22 *	0.19	−0.06	−0.05
Total	T0	−0.16 *	−0.09	−0.08	0.08
	T1	−0.06	−0.01	−0.04	−0.03
	T1/2	−0.19 **	0.04	−0.09	−0.02
	T2	−0.19 **	0	−0.07	−0.01
	Tmean	−0.25 ***	0.01	−0.06	0

T0—time before the procedure (time between administration of pericervical anesthesia and removal of the speculum and insertion of the hysteroscope into the vagina), T1—at the 1st minute of the hysteroscopy, T1/2—mid-procedure of the hysteroscopy, T2—time at the end of the procedure of the hysteroscopy, Tmean—average hysteroscopy procedure time. * *p* < 0.05; ** *p* < 0.01; *** *p* < 0.001.

**Table 14:** Type of hysteroscopy and the ANI.

	Type of Hysteroscopy						U	*p*
	Diagnostic			Operative				
	M ± SD	Min- Max	Me [Q1–Q3]	M ± SD	Min- Max	Me [Q1–Q3]		
T0	59.73 ± 14.24	18–90	60 [50–70]	65.56 ± 15.83	35–100	65.5 [51–78.5]	4806.5	0.01
T1	66.37 ± 15.14	33–96	66 [55–78]	68.16 ± 15.91	34–98	70 [56–80.25]	5553	0.34
T1/2	72.07 ± 13.14	45–99	72 [62–81]	74.97 ± 12.58	43–100	77 [66.75–83]	5148	0.07
T2	69.38 ± 13.25	37–98	70 [60–78]	68.65 ± 12.98	36–96	69.5 [59.75–77.25]	5854	0.76
Tmean	70.5 ± 11.18	42–97	70 [63–79]	70.2 ± 10.78	42–96	69 [62.75–77.25]	5908	0.85

T0—time before the procedure (time between administration of pericervical anesthesia and removal of the speculum and insertion of the hysteroscope into the vagina), T1—at the 1st minute of the hysteroscopy, T1/2—mid-procedure of the hysteroscopy, T2—time at the end of the procedure of the hysteroscopy, Tmean—average hysteroscopy procedure time.

**Table 15:** Type of hysteroscopy and assessment results.

Assessment		Type of Hysteroscopy		Total	χ^2^	*p*
		Diagnostic	Operative			
VAS	0	18 (14.4%)	15 (15.63%)	33 (14.93%)	5.71	0.13
	1–3	66 (52.8%)	36 (37.5%)	102 (46.15%)		
	4–5	25 (20%)	29 (30.21%)	54 (24.43%)		
	6–10	16 (12.8%)	16 (16.67%)	32 (14.48%)		
T0	Normal	98 (78.4%)	76 (79.17%)	174 (78.73%)	0.02	0.89
	Abnormal	27 (21.6%)	20 (20.83%)	47 (21.27%)		
T1	Normal	106 (84.8%)	82 (85.42%)	188 (85.07%)	0.02	0.9
	Abnormal	19 (15.2%)	14 (14.58%)	33 (14.93%)		
T1/2	Normal	121 (96.8%)	93 (96.88%)	214 (96.83%)	0	0.97
	Abnormal	4 (3.2%)	3 (3.13%)	7 (3.17%)		
T2	Normal	117 (93.6%)	90 (93.75%)	207 (93.67%)	0	0.96
	Abnormal	8 (6.4%)	6 (6.25%)	14 (6.33%)		
Tmean	Normal	123 (98.4%)	93 (96.88%)	216 (97.74%)	0.57	0.45
	Abnormal	2 (1.6%)	3 (3.13%)	5 (2.26%)		

T0—time before the procedure (time between administration of pericervical anesthesia and removal of the speculum and insertion of the hysteroscope into the vagina), T1—at the 1st minute of the hysteroscopy, T1/2—mid-procedure of the hysteroscopy, T2—time at the end of the procedure of the hysteroscopy, Tmean—average hysteroscopy procedure time.

**Table 16:** VAS assessment and the ANI.

VAS		N	M ± SD	Min–Max	Me [Q1–Q3]	H	*p*
T0	0	33	60.1 ± 15.13	31–95	61 [50–72]	2.23	0.53
	1–3	102	61.4 ± 14.59	18–94	61 [51–70.8]		
	4–5	54	63.2 ± 16.59	36–100	63 [48.5–76]		
	6–10	32	65.8 ± 14.74	42–92	67 [52.8–75.5]		
T1	0	33	66 ± 17.42	34–98	65 [54–81]	2.35	0.50
	1–3	102	67.2 ± 15.43	33–97	69 [55.3–79]		
	4–5	54	69.2 ± 15.42	40–96	72 [56.3–79.8]		
	6–10	32	64.6 ± 13.64	37–89	66 [56.5–73.8]		
T1/2	0	33	73.2 ± 14.41	43–97	75 [63–85]	0.12	0.99
	1–3	102	73.5 ± 12.74	45–100	73 [64–81]		
	4–5	54	73.5 ± 12.87	49–95	76 [63.3–83]		
	6–10	32	72.6 ± 12.78	46–98	76 [65–80]		
T2	0	33	67.8 ± 15.98	36–98	70 [57–78]	1.68	0.64
	1–3	102	69.6 ± 12.75	40–97	70 [62–78]		
	4–5	54	67.7 ± 11.37	44–92	66.5 [60–76.8]		
	6–10	32	70.8 ± 13.99	47–95	71.5 [58–83.3]		
Tmean	0	33	70.2 ± 13.02	42–96	70 [63–77]	2.05	0.56
	1–3	102	70.6 ± 10.96	46–97	70 [62.3–78]		
	4–5	54	69.1 ± 9.1	54–92	67 [63.3–76]		
	6–10	32	72 ± 11.94	42–94	72.5 [62–82]		

T0—time before the procedure (time between administration of pericervical anesthesia and removal of the speculum and insertion of the hysteroscope into the vagina), T1—at the 1st minute of the hysteroscopy, T1/2—mid-procedure of the hysteroscopy, T2—time at the end of the procedure of the hysteroscopy, Tmean—average hysteroscopy procedure time.

**Table 17:** Relationship between ANI assessment and VAS scores.

VAS		N	M ± SD	Min–Max	Me [Q1–Q3]	U	*p*
T0	Abnormal	47	2.96 ± 2.22	0–8	3 [1–4]	4036.5	0.89
	Normal	174	3.06 ± 2.31	0–10	3 [1–4]		
T1	Abnormal	33	3.24 ± 2.4	0–8	3 [1–5]	2919.5	0.59
	Normal	188	3.01 ± 2.27	0–10	3 [1–4]		
T1/2	Abnormal	7	2.86 ± 2.12	0–6	3 [1.5–4]	730	0.91
	Normal	214	3.05 ± 2.3	0–10	3 [1–4]		
T2	Abnormal	14	2 ±2	0–7	1.5 [0.2–−3]	1022.5	0.06
	Normal	207	3.11 ± 2.29	0–10	3 [1–5]		
Tmean	Abnormal	5	2.2 ± 2.95	0–7	1 [0–3]	392	0.29
	Normal	216	3.06 ± 2.28	0–10	3 [1–4]		

T0—time before the procedure (time between administration of pericervical anesthesia and removal of the speculum and insertion of the hysteroscope into the vagina), T1—at the 1st minute of the hysteroscopy, T1/2—mid-procedure of the hysteroscopy, T2—time at the end of the procedure of the hysteroscopy, Tmean—average hysteroscopy procedure time.

## Data Availability

The data presented in this study are available on request from the corresponding author.
